# The Challenges of Clinical Research in Rare Cancers: Bevacizumab Use in Low-Grade Serous Ovarian and Primary Peritoneal Cancers

**Published:** 2017-05-01

**Authors:** Carolyn Grande, Constance Visovsky, Marcia S. Brose

**Affiliations:** 1 Department of Otorhinolaryngology, Hospital of the University of Pennsylvania Abramson Cancer Center, Philadelphia, Pennsylvania;; 2 University of South Florida College of Nursing, Tampa, Florida

## Abstract

There are challenges to conducting rare cancer clinical trials due to issues surrounding clinical trial design, patient recruitment, and analysis of the study outcomes. This article highlights the challenges of research and data analysis in rare cancers and proposes future study directions using less traditional approaches.

Despite the designation as an uncommon disease, rare cancers exert a significant burden on the health of those affected. Rare cancers are those that have an occurrence rate of fewer than 15 cases per 100,000 individuals or those with fewer than 40,000 new cases per year (National Cancer Institute, 2007). When combined, rare cancers actually account for 27% of all newly diagnosed cancers and 25% of cancer-related deaths (National Cancer Institute, 2007). With the discovery of molecular targets for cancer therapies, commonly occurring cancers, such as breast and lung cancers, can now be subdivided into groups requiring specific treatments to obtain a disease response. These cancer subsets may now meet the criteria for classification as rare diseases.

## RARE CANCERS

Historically, rare cancers have been understudied in clinical research. This may be due in part to the rising costs of drug development in the United States. Consequently, pharmaceutical companies are drawn to concentrating on treatments for more common cancers, which leads to abandonment of treatment identification for patients with rare cancers. To address this, the Orphan Drug Act of 1983 was passed and incorporated benefits to the drug sponsor from the federal government, including enhanced patent protection and marketing rights for development of drugs to treat rare disease (Department of Health and Human Services, 2001). This legislation, to date, has fostered 177 approvals for orphan drug designations to treat rare cancers. Median time from designation to approval was 2.49 years (Stockklausner, Lampert, Hoffmann, & Ries, 2016).

The National Clinical Trials Network (NCTN) is responsible for conducting research that improves outcomes for patients with rare cancers. Rare cancers are thought to be very responsive to treatment, as they have little variability in molecular targets, whereas more common cancers, such as lung cancer, can have hundreds of aberrant pathways, limiting the effects of targeted therapy (Cancer Genome Atlas Research Network, 2012). However, rare cancers pose a particularly difficult issue when it comes to conducting clinical trials due to issues surrounding clinical trial design, patient recruitment, and analysis of the study outcomes.

The intent of this article is to provide a perspective highlighting the challenges of research and data analysis in rare cancers through review of the publication of bevacizumab (Avastin) activity in patients with low-grade serous ovarian and primary peritoneal cancers by Grisham and colleagues (2014).

## OVARIAN CANCER

Epithelial ovarian cancer is the leading cause of death due to a gynecologic cancer in the United States. In 2016, an estimated 22,280 new diagnosis and 14,240 deaths occurred from this neoplasm (Siegel, Miller, & Jemal, 2016). Low-grade serous ovarian cancer (LGSOC) comprises 6% to 8% of all ovarian cancers (Schmeler & Gershenson, 2008).

Previously, the classification of ovarian cancer was identified through a 1 to 3 grading system; however, that practice changed to identification of low-grade serous and high-grade serous ovarian cancers as two distinctive diseases (Kurman, Carcanglu, Herrington, & Young, 2014). In this new identification system, grade 1 and most of grade 2 tumors are reclassified as LGSOC and grade 3 tumors are now identified as high-grade serous tumors.

Low-grade serous ovarian cancer is an indolent cancer with an early-age onset and resistance to cytotoxic chemotherapy with a < 4% response rate (Gershenson et al., 2009). Despite the low response rates to chemotherapy, primary treatment for these tumors consists of surgery plus neoadjuvant or adjuvant chemotherapy. Those with recurrent disease are treated with surgical resection; those with unresectable disease are treated with hormonal therapies, which show a < 9% response rate (Gershenson et al., 2012) or chemotherapy.

Novel systemic options for the treatment of carcinoma have continually advanced over the past several decades. They include targeted therapies such as monoclonal antibodies, tyrosine kinase inhibitors, and multikinase inhibitors. Targeting inhibition of tumor blood vessel development or angiogenesis through administration of bevacizumab has been approved by the US Food and Drug Administration (FDA) for treatment of metastatic colon cancer; nonsquamous non–small cell lung cancer; glioblastoma; metastatic renal cell carcinoma; cervical cancer and platinum-resistant recurrent epithelial ovarian, fallopian tube, or primary peritoneal cancer (Genentech, 2015). Although the results for studies in ovarian cancer have been promising, the vast majority of patients in those studies had a diagnosis of high-grade serous ovarian cancer.

## INFLUENCE OF STUDY DESIGN AND STATISTICAL POWER

In the study by Grisham et al. (2014) discussing the efficacy of bevacizumab in combination with chemotherapy for the treatment of recurrent, low-grade serous ovarian and primary peritoneal cancers, a single-institution, retrospective design was used. Data from 17 patients were collected, but only 15 patients were included in the analysis. Of the 15 evaluable patients, 10 were treated for low-grade serous peritoneal cancer, and the remaining patients had low-grade serous peritoneal cancer or borderline disease. The study outcomes of interest were overall survival and time to disease progression over the 23-week study period.

Statistical tests use data from the study sample to make inferences about a population. In this case, the responsiveness of serous peritoneal cancers to the addition of bevacizumab in treating recurrent disease was studied. Adequate statistical power is needed to be able to detect differences between treatment groups or among the sample that are less likely to be the result of chance. The very small sample size (n = 15) and the retrospective (i.e., observational) nature of the study design limit the statistical power in this study. The easiest way to increase statistical power is to increase the sample size, but this is not realistic in the study of rare cancers, posing a continual issue in obtaining enough statistical power for data analysis.

The precision of the measures used also influences study power. In the study by Grisham et al. (2014), the use of computed tomography (CT) scans as the gold standard is considered a precise measure for the detection of stable, regressive, or progressive disease. However, it is important to note that even standard tests and scans may be interpreted differently among groups of radiologists or oncologists. Therefore, there is a certain amount of standard error in every measure that needs to be considered when interpreting the results (complete response, stable disease, or progressive disease).

Another issue in the study of rare cancers is the potential of differences in treatments and treatment schedules. The heterogeneous nature of the treatments delivered poses difficulty in interpreting the results, as they do not compare the same treatments with each other. In the Grisham et al. (2014) study, 2 patients received bevacizumab alone, and 13 received bevacizumab plus one of the following regimens: paclitaxel, topotecan, oral cyclophosphamide, gemcitabine, or gemcitabine and carboplatin. To complicate matters further, the dosages of bevacizumab varied among the study participants. Bevacizumab was administered in varied doses ranging from 7.5–15 mg/kg, adding further heterogeneity to the sample.

The study results indicate there were no complete responses, six partial responses, five patients with stable disease, and four patients with progressive disease (Table). The survival rates reported in the Grisham et al. (2014) study are consistent with historical 5- to 10-year survival rates for these types of peritoneal cancers, indicating the addition of bevacizumab to chemotherapy for recurrent peritoneal serous cancers may be effective for disease control. However, the treatment variations, when coupled with the small sample size, may influence the perceived benefit of bevacizumab for these types of rare cancers.

**Table:**
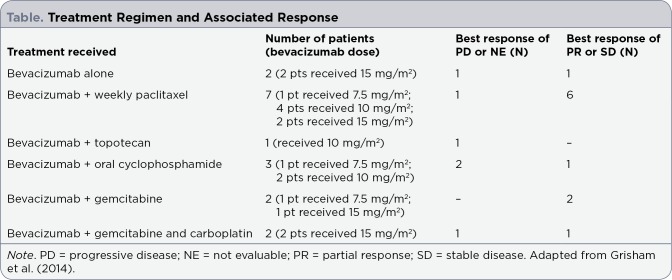
Treatment Regimen and Associated Response

## IMPLICATIONS

Grisham and colleagues’ conduct of a retrospective review is an attempt at information gathering that can inform on treatment outcomes of LGSOC, a very rare cancer that otherwise is not able to be isolated as a single disease entity in a clinical research trial design. As noted, a retrospective review and a small number of patients in a single institution alone are not components that can contribute to practice-changing evidence. In this example, factors of bias in selection, performance, attrition, detection, and random error influence the strength of treatment effects and subsequent clinical applicability.

Existing evidence for rare cancers may be of suboptimal quality due to a paucity of prospective studies and randomized trials and the lowest-level evidence attained through retrospective case reviews and case reports. The challenges of conventional trials in rare cancers are evident. Strategies for research design and interpretation in rare cancers is an area of unmet need. Although progress has clearly been made in terms of orphan drug approvals, the journey forward remains long.

## FUTURE DIRECTIONS

Future clinical trials for the investigation of rare cancers should consider other, less traditional approaches to data analysis. To begin, it is important to note there are limited options for the analysis of these types of data and that the gold-standard approach of a randomized control trial is not possible. Past studies of rare cancers have either lapsed or never opened at all due to low study accrual or lack of potential participants. It is also not possible to use statistical methods to control for many variables (such as age, treatment dose, or schedule) that may impact the outcome of overall survival in such a small sample.

Consideration of open-ended, single-arm studies that are carefully designed and conducted or trials based on genomic characteristics or prospective registry studies is most likely to yield information on best treatment options in rare cancers; however, even they are subject to selection bias and potentially confounding results (Sleijfer & Wagner, 2012). Another consideration is the potential use of targeted therapies for similar genomic anomalies, but they may have different cancers of origin. These are known as "bucket" or "basket" studies and may help accelerate the approval of targeted therapies beyond the initial cancer for which they were developed.

One concern is that some subtypes may or may not be responsive to the targeted therapy based upon differing tumor histology. At present, there are no tumor registries for rare cancers in adults, further limiting knowledge about the best treatment options. However, in 2011, the International Rare Cancers Initiative was established as a partnership between cancer research organizations in the United Kingdom, the United States, and France to facilitate international clinical trials for patients with rare cancers. Through pooling data and conducting meta-analyses of clinical trials, findings concerning subtypes of rare cancers may be enhanced.

It is clear from this and other trials of rare cancers that study design and statistical power are limited using current retrospective clinical trial designs and that new approaches to study design and analysis are needed as well as additional partnerships to make strides in understanding and treating rare cancers.
